# In Situ Electrospinning of “Dry-Wet” Conversion Nanofiber Dressings for Wound Healing

**DOI:** 10.3390/md21040241

**Published:** 2023-04-14

**Authors:** Shanfei Liu, Guilin Wu, Wen Wang, Heng Wang, Yingjun Gao, Xuhong Yang

**Affiliations:** National Engineering Laboratory for Modern Silk, Key Laboratory of Flame Retardancy Finishing of Textile Materials (CNTAC), College of Textile and Clothing Engineering, Soochow University, Suzhou 215123, China

**Keywords:** in situ deposition, handheld electrospinning, nanofiber dressings, “dry-wet” conversion, wound healing

## Abstract

Rapid wound dressings provide an excellent solution strategy for the treatment of wounds in emergency situations. In this study, aqueous solvent-based PVA/SF/SA/GelMA nanofiber dressings fabricated by a handheld electrospinning device could deposit quickly and directly on the wound, perfectly fitting wounds with various sizes. Using an aqueous solvent overcame the disadvantage of using the current organic solvents as the medium for rapid wound dressings. The porous dressings had excellent air permeability to ensure smooth gas exchange at the wound site. The distribution range of the tensile strength of the dressings was 9–12 Kpa, and the tensile strain was between 60–80%, providing sufficient mechanical support during wound healing. The dressings could absorb 4–8 times their own weight in solution and could rapidly absorb wound exudates from wet wounds. The nanofibers formed ionic crosslinked hydrogel after absorbing exudates, maintaining the moist condition. It formed a hydrogel–nanofiber composite structure with un-gelled nanofibers and combined the photocrosslinking network to maintain a stable structure at the wound location. The in vitro cell culture assay indicated that the dressings had excellent cell cytocompatibility, and the addition of SF contributed to cell proliferation and wound healing. The in situ deposited nanofiber dressings had excellent potential in the urgent treatment of emergency wounds.

## 1. Introduction

As one of the largest organs of the human body, most skin tissues are in direct contact with the external environment and have functions like barrier protection, material and information exchange, regulating body responses, and sensing external stimuli [1,2,3]. Meanwhile, due to its fragility, the skin is vulnerable to damage in the form of wounds, losing the properties and functions of the skin and leading to a metabolic disorder of the wound area or even damage to the body [4]. Therefore, wound healing is essential for maintaining human health [5]. Wound healing is a complex and continuous process that requires an appropriate environment [6]. Wound dressings, as good substitutes for the damaged skin at the wound site, can temporarily provide the skin-like function for the wound site. Therefore, wound dressings have become one of the most important treatment methods in clinical wound treatment [7,8]. Wound dressing materials like nonwovens [9], gels [10], sponges [11], hydrogels [12], membranes [13], and nanofiber membranes [14] that have good flexibility, stability, and biodegradability while maintaining wound wetness, hemostasis, and the ability to absorb exudates are becoming hot research topics.

Electrospinning is an efficient method that can produce nanofibers at a low cost and on a continuous scale [15]. Electrospun nanofibers have broad development space and practical value in the fields of biomedicine because of their characteristics of a large specific surface area, small pore size, and controllable deposition density [16,17,18,19,20]. Electrospun nanofiber dressings can form barriers that protect the wound from external factors; meanwhile, they possess good permeability, faster hemostasis, and other properties [21,22,23]. In the practical application process, the traditional electrostatic spinning equipment is quite bulky, leading to limitations in the flexibility and practicality of fiber deposition [24], which limits their wider application in wound healing, especially in outdoor first aid, rapid wound management, and wound hemostasis. The integrated, battery-operated electrospinning apparatus based on a miniaturization and the in situ deposition electrospinning strategy provide a good solution to the limitations of conventional electrospinning [25]. Jiang et al. first used the airflow-directed in situ electrospinning method to prepare the n-octyl-2-cyanoacrylate nanofibers, which achieved rapid large-area hemostasis of viscera within tens of seconds [26]. Liu et al. prepared in situ electrospun CuS composite nanofibers using a portable electrospinning device, which possessed better compactness on rough wound surfaces, resulting in better rapid hemostasis and ablation of superbugs [27]. Zhang et al. directionally deposited N-octyl-2-cyanoacrylates nanofibers onto the living organ during a minimally invasive surgery, stopping bleeding in 5 s [28]. Ding et al. designed a portable electrospinning device with multi-functions and prepared skin-like flexible nanofiber membranes with high water resistance and permeability composed of ethanol-soluble polyurethane and fluorinated polyurethane [29]. Tang et al. used a handheld electrospinning device to prepare nanofiber dressings, with photodynamic therapy effect, which were perfectly suited for wounds of different sizes [30]. Portable electrospinning based on an in situ deposition principle provided a very timely and effective method for wound treatment, combining biopolymer materials, drug carriers, etc. to endow nanofiber dressings with multiple functions, such as antibacterial, antiviral, immune regulation, and promoting healing [31]. However, polymers currently used for handheld electrospinning require organic reagents to dissolve, and incompletely volatile organic solvents can cause toxicity to wound tissue. Therefore, it is necessary to design polymer spinning systems without organic solvents, such as water as a solvent.

Alginate, as a polysaccharide compound extracted from marine plants, possess good biocompatibility, biodegradability, and easy formability. The biomaterials prepared by alginate have a wide range of applications in biomedicine [32]. Due to its good liquid absorption, angiogenesis, antiallergy, antimicrobial, anti-inflammatory, antioxidant, immune regulation, and hemostatic properties, alginate-based biomaterials have become an important component of wound dressing [33,34,35]. Due to the characteristics of alginate like a high surface tension, high conductivity, and a lack of chain entanglement, the alginate solution cannot form nanofibers stably or stably in a high voltage electric field. Meanwhile, the mechanical properties of alginate nanofibers are poor, which limits their application in handheld electrospinning dressings [36,37]. The problem of poor resistance in alginate electrospinning can be solved by mixing polymer with macromolecular chains of strong hydrogen bonds and good entanglement. One of the more commonly used polymers is polyvinyl alcohol (PVA), which is a water-soluble polymer with excellent electrospinning spinnability and is beneficial for wound healing [38,39,40]. For better wound healing, silk fibroin (SF) is often incorporated into dressing systems because of their good healing-promoting qualities, excellent biocompatibility, and good adsorption of wound exudates to mimic the ECM [41,42]. Thanks to the optical cross-linking network of gelatin methacryloyl (GelMA), nanofiber membranes containing GelMA have better mechanical strength, which can solve the problem of the low strength of nanofiber dressings [43,44]. The concept of healing is divided into dry healing and wet healing according to the different wetness of wound conditions. Previous studies indicate that moist wounds are beneficial for wound healing [45]. However, the moist condition is prone to bacterial growth, leading to an infection of the wound. The excessive moist environment causes the wound edge impregnation, edema, and infection, which leads to the functional damage of the skin around the wound, thus affecting the wound healing. Therefore, it is essential to create a wound healing environment according to the situation of the wound and maintain the dry/wet balance of the wound site for wound healing [46]. For dry wounds with less wound exudates, keeping the wound surface slightly moist and preventing the invasion of external microorganisms help the wound heal. For moist wounds with more exudates, removing the wound exudates while maintaining the wet wound environment is conducive to wound healing. However, the in situ deposition nanofiber dressings prepared at present are difficult to adapt to a variety of wound types, which limits their applications in wound healing. 

In this work, we developed the nanofiber dressings made of PVA/SF/SA/GelMA by a handheld electrospinning device based on an in situ deposition principle. The morphology structure, chemical structure, liquid absorption performance, “dry-wet” conversion properties, and cytocompatibility of the nanofiber dressings were characterized to investigate its potential application in wound healing. Meanwhile, the nanofiber dressings with different sodium alginate (SA) solution concentrations were prepared and characterized to explore the effect of SA characteristics on the preparation and properties of the nanofiber dressings. 

## 2. Results and Discussions

### 2.1. Fabrication and Morphological Characterization of the Nanofiber Dressings

**Scheme 1:**
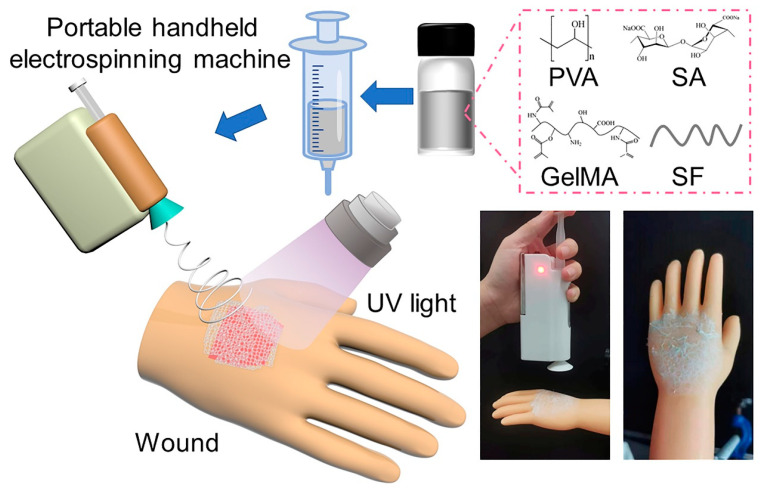
Schematic illustration of the fabricating PVA/SF/SA/GelMA deposited nanofiber dressings.

Scheme 1 showed the schematic diagram of the preparation process of the nanofiber dressing. The preparation of nanofiber dressings by an in situ deposition method was convenient and controllable, and the spinning parameters could be adjusted according to the position, type, and exudates of the wound to prepare more suitable dressings for the wound and promote the wound healing. In this study, the voltage we used was set to 10 kV, the receiving distance was 15 cm, and the flow rate was 0.4 mL/h. For the mixed aqueous solution, the content of SA has a great influence on the spinnability of its electrospinning, so the nanofibers with SA solutions of different concentrations (4.0, 3.0, 2.5, 2.0, 1.5 wt.%) were studied. 

**Figure 1:**
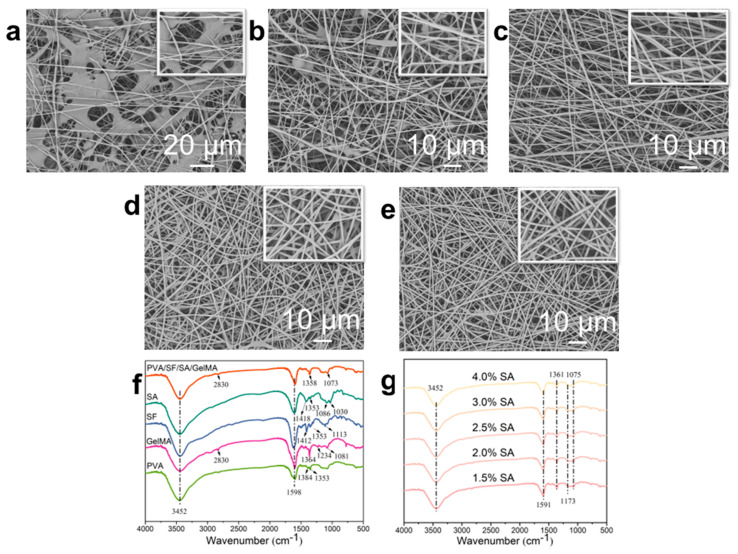
SEM images of the deposited nanofiber dressings with SA solutions of (**a**) 4.0 wt.%, (**b**) 3.0 wt.%, (**c**) 2.5 wt.%, (**d**) 2.0 wt.%, (**e**) 1.5 wt.% SA concentrations. (**f**) The FTIR curves of the raw polymers and the nanofiber dressings. (**g**) The FTIR curves of the nanofiber dressings with SA solutions of different SA concentrations.

The apparent morphology of nanofibers prepared from SA solutions with different SA concentrations was studied by observing scanning electron microscopy (SEM) images (Figure 1a–e) to evaluate the spinnability of the mixed solution. The properties of the solution, including viscosity and conductivity, as well as the spinning process parameters, such as reception distance and spinning voltage, have a significant impact on the morphology of the fibers [47]. It can be seen from Figure 1a that there were a large number of polymer agglomerations in the nanofiber dressings prepared by SA solution with 4.0 wt.% concentration; the nanofibers morphologies were irregular, and the surface was relatively rough. At the same time, there were beading, merging, and splitting structures in some nanofibers. When the concentration of SA solution was 3.0 wt.%, the polymer agglomeration disappeared, the nanofibers developed into regular morphology, and the smoothness of the nanofibers’ surface increased, but there were still beaded, parallel, and split structures in the nanofiber (Figure 1b). This is mainly due to the inability of the mixed solution to form nanofibers smoothly and stably in the high voltage electric field because of the characteristics of high surface tension, high conductivity, and lack of chain entanglement of SA when the concentration is high [36,37]. With the decrease in SA content in the mixed solution, its influence on the spinning performance weakened. Therefore, as the concentration of SA solution decreased, the regularity of the nanofibers morphology improved, and the beading, merging, and splitting structures in the nanofibers gradually disappeared, forming the nanofiber dressings’ structure with a smooth surface, regular morphology, and homogeneous distribution (Figure 1c–e). These results showed that the spinnability of the spinning solution increased with the decrease in the concentration of SA. 

Figure 1f,g showed the Fourier transform infrared spectrometer (FTIR) spectra of the raw polymers and the nanofiber dressings prepared of PVA/SF/SA/GelMA with SA solutions of different SA concentrations to explore the functional group structure, which was closely related to the liquid absorption performance of the dressings. The polymers used in the preparation of nanofibers contained a large number of functional groups such as O-H, C-H, C-O, and C=O. In the macromolecules of the final prepared PVA/SF/SA/GelMA nanofiber dressings, the positions and intensity of the functional group peaks in various compounds interacted with each other. In the FTIR curve of the nanofiber dressings, there were wide strong peaks between 3200 and 3500 cm^−1^, which were caused by the vibration of O-H and N-H groups. The peak near 1598 cm^−1^ was formed by the vibration interaction of N-H, C-H, and C=O functional groups. The bending vibration of C-H group forms a characteristic peak near 1358 cm^−1^, and the characteristic peak caused by a C-O group’s vibration near 1073 cm^−1^. The FTIR spectra of nanofibers prepared with SA solutions of different SA concentrations had the similar frame and characteristic peaks, indicating that the concentration of SA had no obvious effect on the chemical structure of the PVA/SF/SA/GelMA nanofibers. 

**Figure 2:**
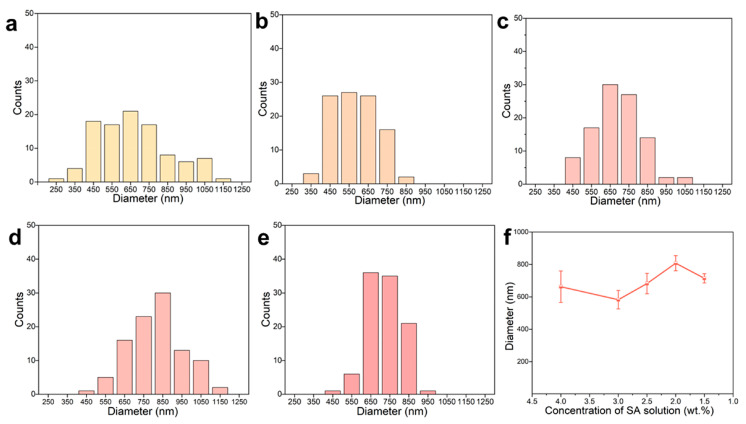
Statistical count of the diameter of the deposited nanofiber dressings with SA solutions of (**a**) 4.0 wt.%, (**b**) 3.0 wt.%, (**c**) 2.5 wt.%, (**d**) 2.0 wt.%, (**e**) 1.5 wt.% SA concentrations. (**f**) Average diameter variation curve of nanofiber dressings with SA concentrations.

Figure 2 illustrated the diameter distribution of PVA/SF/SA/GelMA nanofiber dressings prepared with SA solutions of different SA concentrations. The diameter and distribution of nanofiber dressings were greatly affected by the spinning stability. Therefore, the variation of SA concentration had significant impact on the diameter and distribution of nanofibers. Due to the poor spinnability and low spinning stability, the nanofiber dressings prepared with SA solution containing 4.0 wt.% SA possessed poor uniformity, resulting in a wide range of diameter distribution: 250~1050 μm. At the same time, the electrospinning process was unstable, resulting in jet streams splitting into multiple streams, producing more nanofibers with a smaller diameter. With the decrease in SA content, the spinning stability was improved, the nanofiber shape and diameter gradually became uniform, and the difference between nanofiber diameters became smaller, and the diameter distribution range gradually decreased. For the nanofibers prepared with SA solutions of 3.0, 2.5, 2.0, and 1.5 wt.% SA, their diameter distribution ranges were 450~850, 550~850, 650~850, and 650~850 μm, respectively. Compared to the honey loaded alginate/PVA nanofibers, the PVA/SF/SA/GelMA nanofibers had a tighter and more uniform diameter distribution and smoother surface structure [40]. According to the surface morphology, apparent structure, and diameter distribution of the nanofiber dressings, SA solutions containing 2.5, 2, and 1.5 wt.% could be used for the stable and continuous fabrication of the nanofiber dressings and to prepare nanofiber dressings with a uniform structure. 

### 2.2. Porous Structure and Moisture Absorption Performance of the Nanofiber Dressings

**Figure 3:**
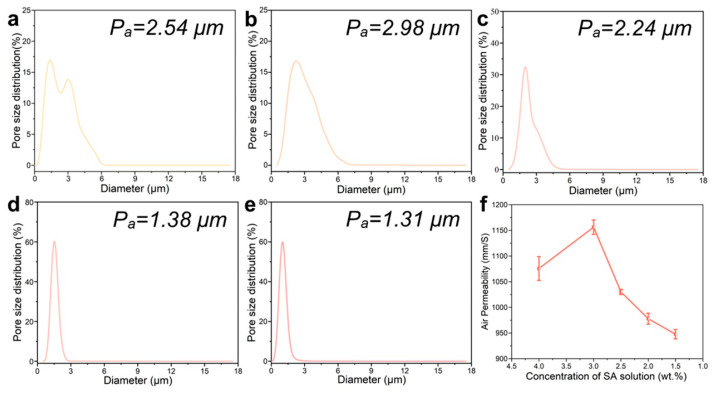
Pore size distribution of the deposited nanofiber dressings with SA solutions of (**a**) 4.0 wt.%, (**b**) 3.0 wt.%, (**c**) 2.5 wt.%, (**d**) 2.0 wt.%, (**e**) 1.5 wt.% SA concentrations. (**f**) Air permeability variation curve of nanofiber dressings with SA concentrations.

The porous structure was investigated to evaluate the air permeability and moisture absorption of the nanofiber dressings. The pore size and distribution of the nanofiber dressings with SA solutions of different concentrations were shown in Figure 3a–e. The mean pore size of the nanofiber dressings gradually increased from 2.54 μm to 1.31 μm. The pore size distribution ranges also changed significantly. The pore size distribution of the nanofiber dressings with 4.0 wt.% SA concentration was in a large range of 1.2~4.8 μm. From its apparent structure, it could be seen that the existence of a polymer agglomerate structure destroyed the connecting hole formed by nanofiber stacking, resulting in a larger gap in pore size. At the same time, due to less nanofiber stacking, the pore size became larger. With the decrease in SA content, the spinning stability increased, the formed nanofiber morphology tended to be stable, the arrangement of nanofiber tended to be uniform, the pore size distribution range gradually shrunk, and the nanofiber bulk density increased, making the pore size smaller.

In the process of wound healing, it is important to ensure the smooth exchange of gas at the wound site, so the permeability of the dressings is an important index to evaluate the performance of the dressings [48]. As shown in Figure 3f, for the nanofiber dressings with 4.0 wt.% SA concentration, the existence of a polymer agglomerate structure destroyed the gas exchange channel, but the large pores made it have good air permeability. For the nanofiber dressings prepared with an SA solution of 3.0 wt.% SA, the polymer had less agglomerate structure and a higher pore size, which improved its air permeability. With the increase in nanofiber stacking density, the decrease in pore size, and the increase in buckling pore size of nanofibers, the decrease of directly connected pore size, the air permeability of nanofiber dressings gradually declined. 

**Figure 4:**
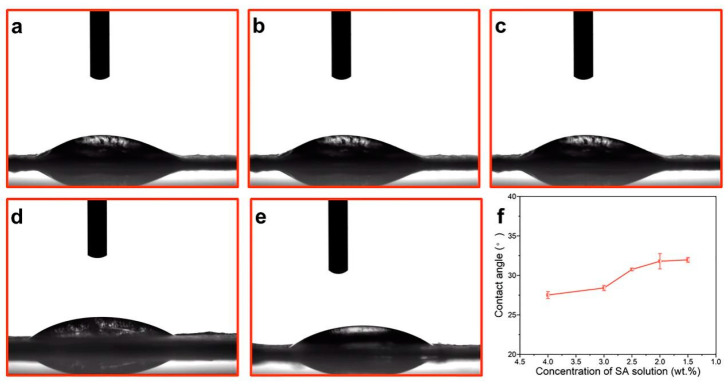
The contact angles of the deposited nanofiber dressings with SA solutions of (**a**) 4.0 wt.%, (**b**) 3.0 wt.%, (**c**) 2.5 wt.%, (**d**) 2.0 wt.%, (**e**) 1.5 wt.% SA concentrations. (**f**) Contact angle variation curve of nanofiber dressings with SA concentrations.

The water contact angles of the nanofiber dressings were investigated to evaluate the absorption performance of wound exudates during the wound healing process. For hydrophilic materials, the contact angle decreases with the increase in solution absorption. As the surface roughness increases, the hydrophilicity of the material surface increases [49]. The contact angles and the variation curve of the nanofiber dressings were illustrated in Figure 4. The contact angle increased slightly with a decreasing SA concentration from 31.96° to 27.51°, which indicated that the nanofiber dressings prepared with SA solution of a higher SA concentration had better hydrophilicity. This was mainly due to the excellent hydrophilicity of SA and the rougher surface structure of the nanofiber dressings prepared with an SA solution of a higher SA concentration. The difference in contact angle of the nanofiber dressings decreased as the difference in SA concentration decreased and the stability of the surface structure of the prepared nanofiber dressings increased.

### 2.3. Mechanical Properties of the Nanofiber Dressings

**Figure 5:**
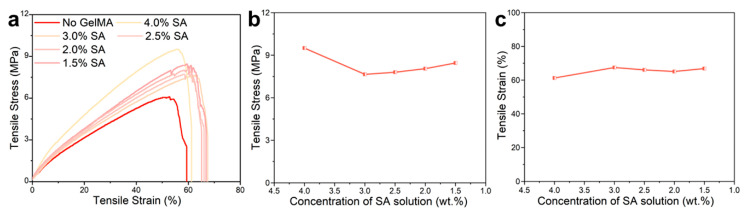
(**a**) Strain/stress curves of the nanofiber dressings with SA solutions of different SA concentrations and PVA/SF/SA with 2.5 wt.% SA concentration. (**b**) Tensile stress variation curve and (**c**) Tensile strain variation curve of nanofiber dressings with SA concentrations.

As the substitute for healthy skin, wound dressings should have mechanical properties similar to that of healthy skin (tensile strength: 1–20 MPa, tensile strain: 30–70%) [50]. The strain/stress curve of the nanofiber dressings were shown in Figure 5. Compared with the strain/stress curve of nanofiber dressings prepared from solutions without GelMA addition (SA: 2.5 wt.%), the nanofiber dressings prepared from PVA/SF/SA/GelMA solutions had 35% higher tensile stress and 32% longer tensile strain, indicating that the photo-crosslinked network of GelMA significantly enhanced the mechanical properties of the prepared nanofiber dressings. The analysis of these curves showed that the tensile stresses of all samples were in the range of 6–10 MPa, and the tensile strain ranges were 60–70%, which were within the required ranges. Therefore, the nanofiber dressings met the mechanical performance requirements and had excellent biomechanical compliance to use as the substitute for healthy skin in the clinical treatment of wounds. At present, the reported tensile strength of PVA/SA electrospun nanofiber membranes are generally within the range of 2–5 MPa [39,51]. The nanofiber membrane prepared with the PVA/SF/SA/GelMA solution system had better mechanical properties. The 3D disarrangement of electrospun nanofibers results in the preparation of nanofiber materials with good anisotropy [52]. The nanofibers were rearranged along the direction of the force under the external tensile force, resulting in a significant increase in tensile stress. The cohesion force in the nanofibers and the entanglement force and friction force between the nanofibers were the main forms of tensile force for the nanofiber dressings. For the nanofiber dressings prepared with an SA solution of 4.0 wt.% SA concentration, its tensile stress was higher, which was mainly because the polymer agglomeration enhanced the cohesion force of the nanofibers, but at the same time, it caused the rearrangement of the nanofibers along the direction of the tensile force and the reduction of the nanofiber slip, resulting in a low tensile strain. With the increase in the spinnability of the polymers and the homogeneity of the prepared nanofibers, the tensile stress decreased and the tensile strain increased slightly. There was no significant difference in the tensile stress and tensile strain of the nanofiber dressings prepared with an SA solution of different SA concentrations, which indicated that the effect of SA concentration on the mechanical properties of the nanofiber dressings was small.

### 2.4. “Dry-Wet” Conversion of the Nanofiber Dressings

**Figure 6:**
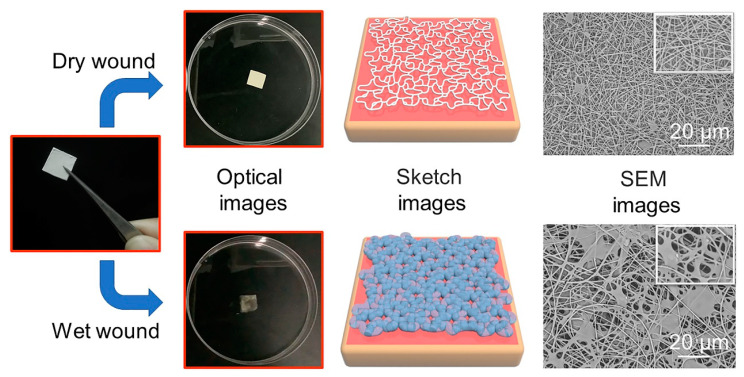
Optical images, sketch images and SEM images of the nanofiber dressings on dry and wet wound conditions.

The metal ion chelating property of SA made nanofibers chelate with a large number of divalent metal ions contained in an SBF solution to form metal ion alginate materials [32]. When the nanofiber dressings were in contact with the wound surface, the metal cation ions in the wound exudates, such as Ca^2+^ and Mg^2+^, exchanged with the Na^+^ in the nanofiber dressings and chelated to form insoluble metal alginate, which absorbed the solution and swelled to form a hydrogel. While absorbing a large amount of wound exudates, the crosslinked macromolecule could maintain the stable 3D structure. Meanwhile, the photo-crosslinked structure formed by GelMA macromolecules could also increase the structural stability of the hydrogel. Based on the amount of exudates produced by the wound, the wound can be divided into dry wound and moist wound categories [46]. The optical, sketch, and SEM images of the deposited nanofibers on a dry and wet wound were shown in Figure 6. On the dry wound surface, the nanofiber dressings had a low degree of ion-exchange and gelation; the dressings maintained their original 3D fiber state without a hydrogel structure, providing physical protection against an external contamination of the wound and maintaining a small amount of exudates. For the wet wound surface, a large amount of ion exchange produced a hydrogel, which promptly transferred excess wound exudates and was stored in the hydrogel structure, providing a moist healing condition while preventing exudates from infecting healthy skin. At the same time, the other polymer components did not gel and swell. The un-gelled nanofibers were used as the reinforcement to combine with the hydrogels to form the hydrogel–nanofiber composite structure, which had strong mechanical properties, providing the necessary mechanical and structural support for the hydrogel–nanofiber composite structure. The “wet-dry” conversion characteristics and the formed hydrogel–nanofiber composite structure made the nanofiber dressings have good liquid absorption performance and overcome the shortcomings of easy structural damage. 

**Figure 7:**
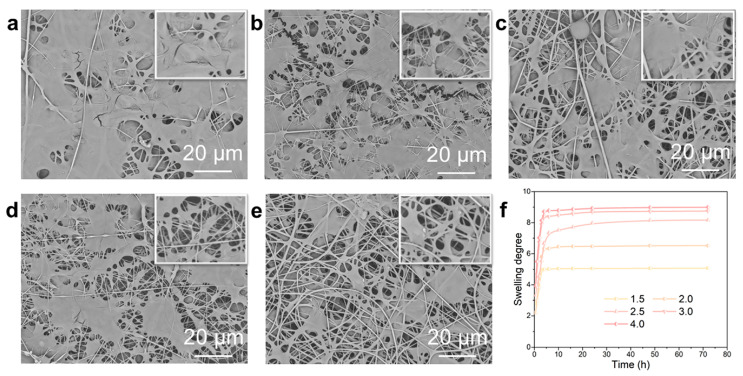
SEM images of the deposited nanofiber dressings with SA solutions of (**a**) 4.0 wt.%, (**b**) 3.0 wt.%, (**c**) 2.5 wt.%, (**d**) 2.0 wt.%, (**e**) 1.5 wt.% SA concentrations after swelling. (**f**) The swelling degree curves of the nanofiber dressings with SA solutions of different SA concentrations.

The SEM images in Figure 7a–e demonstrated the composite structure of the nanofiber dressings prepared with SA solutions of different SA concentrations after swelling. The gel-swelled nanofiber fraction formed polymer aggregates, and there were different binding modes, which were mainly due to the irregular arrangement of nanofibers in the deposited dressings. The gelled nanofibers bound to each other in the form of polymer aggregates, making the gelled dressings more compact, and the bonded gelled nanofibers reduced the pore size and their distribution range. As the SA content increased, the ion-exchange increased, and the generated hydrogel fraction increased, which made the hydrogel aggregation fraction in the nanofibers increase, and their proportion in the hydrogel–nanofiber composite structure gradually increased.

The swelling degree curves of the nanofiber dressings with different SA concentrations were shown in Figure 7f. The nanofiber dressings absorbed SBF and swelled quickly, reaching the maximum swelling degree in a short time and keeping stable. All the fabricated nanofiber dressings absorbed a large amount of wound exudates, and their swelling rates were 5–9 times their own weights. The liquid absorbed by swelling could be divided into liquid partially absorbed by nanofibers, liquid partially absorbed by hydrogels, and liquid stored in the pores of nanofibers. With the increase in SA concentration, the swelling rate gradually increased. Meanwhile, the nanofibers contained a large amount of polar groups, which made them have excellent solution absorption [29,31].

### 2.5. Cytocompatibility of the Nanofiber Dressings

For the construction of wound dressings and materials that promote wound healing, biocompatibility is a key indicator [53]. Most of the reported in situ deposition electrospinning nanofiber dressings use organic compounds as solvents within the system [25,26,27,28,29,30,31]. Compared to silk fibroin materials prepared from aqueous solutions, silk fibroin materials prepared from a formic acid solution as a solvent have poor cell compatibility [54], indicating that, compared to organic reagent spinning systems, water as a solvent can minimize wound damage. In this system, PVA/SF/SA/GelMA only required water as the solvent, so it had better cell compatibility compared to existing in situ deposition electrospun nanofiber dressings. The proliferation and spreading morphology of L929 cells on the PVA/SF/SA/GelMA nanofiber dressings through CCK-8 and a cell staining assay were investigated to evaluate the cytocompatibility of the nanofiber dressings.

**Figure 8:**
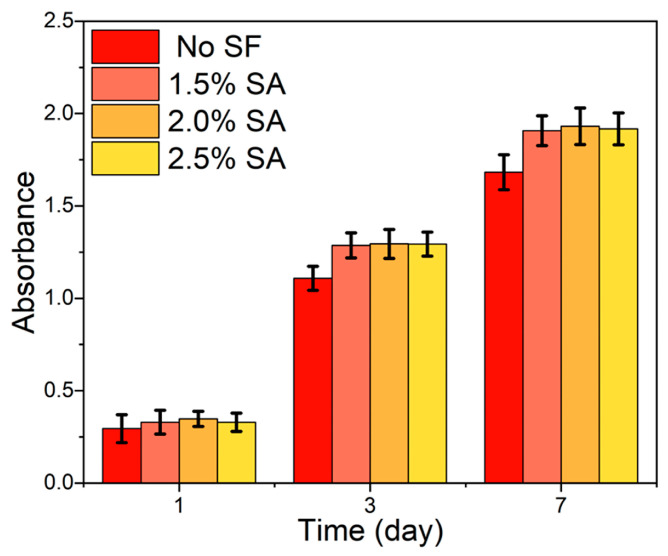
CCK-8 assay results illustrating the proliferation of L929 cells cultured with the deposited nanofiber dressings.

The CCK-8 assay results were shown in Figure 8 to evaluate the proliferation of L929 cells cultured with the fabricated nanofiber dressings. With the increase in culture time, the absorbance of the samples with and without SF increased obviously, indicating that all the fabricated dressings possessed better cytocompatibility and could promote cell growth and proliferation. The absorbance of L929 cells cultured with the PVA/SF/SA/GelMA nanofiber dressings was higher than that of L929 cells cultured with the PVA/SA/GelMA nanofiber dressings (SA: 2.5 wt.%), and with the increase in culture time, the high amplitude increased steadily, which indicated that SF had better cytocompatibility to promote cell proliferation and could promote better and faster wound healing. Moreover, there was no significant difference between the absorbances of L929 cells cultured with PVA/SA/GelMA nanofiber dressings with SA solutions of different SA concentrations, which indicated that the different SA contents had no significant effect on the cytocompatibility of the fabricated nanofiber dressings. 

**Figure 9:**
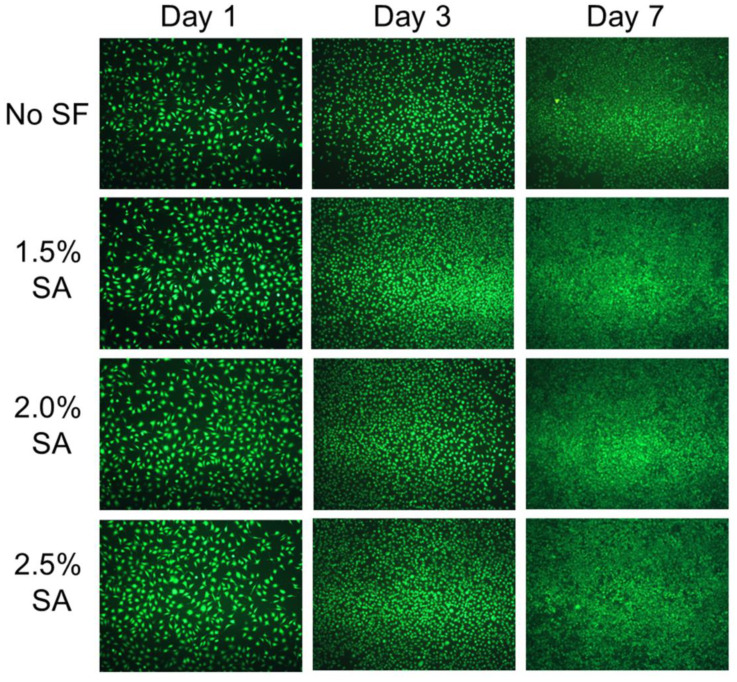
Fluorescence microscopic morphology of L929 cells cultured with the nanofiber dressings.

The L929 cells were cultured with the PVA/SF/SA/GelMA nanofiber dressings for 1, 3, and 7 days. The live cells were stained with calcein-AM and observed with a fluorescence microscope to analyze the proliferation and spreading morphology of L929 cells, and the results were shown in Figure 9. After one day of culture, the cells attached well and presented good morphology, but there was no significant proliferation and significant cell spreading and aggregation. With the increase in culture time, the number and spread area of cells increased significantly and showed a trend of gradual aggregation with good cell morphology. On the seventh day of cell culture, compared with the initial stage of cell culture, the aggregation between cells increased significantly. These results showed that the PVA/SF/SA/GelMA nanofiber dressings could promote cell growth, proliferation, and spread. Compared with the nanofiber dressings without SF, the cells cultured with the nanofiber dressings containing SF had more proliferating cells, spreading area, and aggregation morphology, which indicated that the addition of SF was conducive to the growth and proliferation of cells. There was no significant difference in the number of cell proliferation, spreading area, and aggregation morphology of cells cultured with the PVA/SF/SA/GelMA nanofiber dressings with SA solutions of different SA concentrations, which showed that the concentration of SA solution had no significant effect on cell proliferation and spreading. These results were consistent with the results of the CCK-8 assay.

## 3. Materials and Methods

### 3.1. Materials

PVA (1788, 90%), Alginate acid sodium salt (alginate from brown algae, low viscosity) were purchased from Sigma-Aldrich, (Merck KGaA Co., St. Louis, MO, USA). SF (average Mn ≧ 100,000) was purchased from Simatech Biotechnology Co., Ltd. (Suzhou, China). GelMA with a degree of amino substitution of 60% was purchased from Engineering For Life Co., Ltd. (Suzhou, China). The reagents used in cell culture in vitro were purchased from Gibco (Thermo Fisher Scientific Co., Waltham, MA, USA). Cell Counting Kit (CCK8) and the cell viability (Calcein-AM) test kit were provided by KeyGEN BioTECH Co., Ltd. (Nanjing, China). 

### 3.2. Preparation of PVA/SF/SA/GelMA Nanofiber Dressings

Based on the different dissolution processes of different polymers, it was necessary to pay attention to the order of adding polymers when configuring the mixing solution. After many pre-experiments, we found the polymer addition sequence with better solubility. First, 10 g PVA and 10 g SF were dissolved in 100 mL water, respectively, and PVA and SF solution with concentration of 10 wt.% were prepared after magnetic stirring for 8 h. These two solutions were mixed to prepare PVA/SF solution with a ratio of 10:7. Second, a certain amount of SA was added in 100 mL water to prepare uniform SA solution by stirring for 8 h. The SA solution was added to the PVA/SF solution to prepare PVA/SF/SA mixed solution after stirring. The electrospinning PVA/SF/SA/GelMA solution was prepared by adding GelMA with a concentration of 10 wt.% to the PVA/SF/SA mixture solution after stirring and ultrasonic treatment. The prepared solution was deposited in situ by a portable handheld electrospinning machine (JUNADA Technology Co., Ltd., Qingdao, China) with a 20-gauge flat head stainless steel needle to form PVA/SF/SA/GelMA nanofiber dressings. 

### 3.3. Characterizations

#### 3.3.1. Morphology and Structural Characterization

The surface morphology structure of the samples was observed by a scanning electron microscope (SEM, Hitachi, S8100, Tokyo, Japan) at an acceleration voltage of 10 kV. The chemistry structure of the samples was measured by a Nicole iS50 FTIR spectroscopy (Thermo Fisher Scientific, Nicolet Nexus 670, Waltham, MA, USA) in the attenuated total reflection mode (ATR). The absorption spectral range was 500–4000 cm^−1^. The ImageJ software (ImageJ 1.48, National Institutes of Health Co., Bethesda, MD, USA) was used to measure and count the diameter of nanofibers in the SEM images, and the measured quantity was 300 pieces per image.

#### 3.3.2. Porous Structure and Moisture Absorption Performance Test

The porous structure of the samples, including the pore size and the distribution, was analyzed with Porometer 3G zh (Quantachrome Instruments, Boynton Beach, FL, USA) based on the capillary flow porosimetry. The contact angles of the samples were measured with a Contact angle tester (OCA15EC, Data Physics Instruments Co., Filderstadt, Germany) to characterize the hydrophilicity of the samples. The samples with size of 5 cm × 5 cm (weight as W_0_) were immersed in simulated body fluid (SBF) at 37 °C for 0.5, 1, 2, 4, 6, 8, 12, and 24 h; then, the samples, after swelling, had the residual solution removed and weighted (W_1_). The swelling degree (g/g) was calculated by (W_1_–W_0_)/W_0_. The air permeability of the samples was tested on the YG461E/II digital permeability meter (Fangyuan Instrument, Wenzhou, China). The test pressure difference was set as 100 Pa/mm H_2_O. Five valid data were measured for each sample, and the average value was taken.

#### 3.3.3. “Dry-Wet” Conversion Test

The samples with the size of 5 cm × 5 cm were immersed in a small amount of SBF and a large amount of SBF, respectively. A small amount of SBF could wet the surface of the samples, and a large amount of SBF could completely immerse the samples. After 24 h, the swollen samples were taken out. After freeze-drying, the surface morphology and structure of the swollen nanofiber dressings were observed with SEM to study the change in structure and morphology of the deposited dressings in dry and wet wounds. 

#### 3.3.4. Tensile Mechanical Properties Testing

The tensile mechanical properties of the nanofiber dressings were analyzed by an electromechanical universal testing machine (WDW-20; Shanghai Hualong Test Instruments Co., Shanghai, China); the gauge length was set as 10 cm with a stretching speed of 50 mm/min.

#### 3.3.5. Cell Culture and Cytocompatibility Test

The sterilized samples were cut into a circle with a diameter of 14 mm and placed in 24-well cull culture plates. The mouse lung fibroblast cells (L929 cells, 10^5^ cells/well, Cell Bank of the Chinese Academy of Science, Shanghai, China) were seeded on the surface of the samples and cultured in medium composed of 90 *v/v* % high glucose Dulbecco’s modified Eagle’s medium (DMEM), 10 *v/v* % fetal bovine serum (FBS), and 1 *v/v* % penicillin/streptomycin. The plates were placed in a cell incubator with 37 °C and 5% CO_2_ for culturing for 1, 3, and 7 days. The cytotoxicity of the samples was measured by CCK-8 assay. Cells were cultured at each time point, the medium was removed and washed by PBS, CCK-8 test solution (400 μL/well) was added, and cells continued to incubate in dark conditions for 4 h. The supernatant was measured by a microplate reader (Multiskan GO, Thermo, Waltham, MA, USA) at 450 nm to investigate the absorbance value (O.D. Value) of the cultured cells. The growth, proliferation, and spreading of the cells were observed by confocal laser scanning microscope (CLSM, LSM 700, Carl Zeiss, Oberkochen, Germany) based on the Calein-AM staining method.

## 4. Conclusions

In this study, based on the principle of in situ deposition, PVA/SF/SA/GelMA nanofiber dressings for promoting wound healing were developed using handheld electrospinning equipment. In situ deposition electrospun nanofibers can directly cover the wound site and can adapt to different wound sites and shapes. Compared with traditional electrospinning, it can provide more compact coverage and better comfortability for the wound. The pore size analysis and SEM images showed that the nanofiber dressings had a porous structure to provide good air permeability to the wound. The tensile test results showed that the nanofiber dressings had mechanical properties similar to skin. Photo-crosslinking between GelMA macromolecular chains increased the mechanical properties of the nanofiber dressings. For dry wounds with little wound exudates, the nanofiber dressings provided good protective properties for the wound. The metal ion chelating property of SA allowed the nanofiber dressings to exchange with the metal ions in the wound exudates and form a hydrogel after swelling. The ion crosslinked alginate hydrogel and photo-crosslinked GelMA hydrogel to form an ion crosslinked–photo-crosslinked composite hydrogel. Meanwhile, the un-gelled nanofibers maintained the nanofiber structure and were used as reinforcement to form a hydrogel–nanofiber composite structure with a hydrogel, providing moist healing conditions for the wound while providing excellent mechanical and structural support for the nanofiber dressings. The results of an in vitro cell culture assay showed that the nanofiber dressings possessed excellent cytocompatibility and the incorporation of SF promoted cell growth and proliferation. In summary, the nanofiber dressings possessed excellent absorption properties, skin-like mechanical performance, and excellent cytocompatibility. At the same time, the unique “dry-wet” conversion properties formed a hydrogel–nanofiber composite structure that provided excellent conditions for wound healing. The handheld electrospinning device and in situ deposition principle provide an excellent solution strategy for the timely treatment and healing of wounds, especially in an emergency.

## Data Availability

Data are contained within the article.
